# Automatic gait EVENT detection in older adults during perturbed walking

**DOI:** 10.1186/s12984-025-01560-9

**Published:** 2025-02-28

**Authors:** Shuaijie Wang, Kazi Shahrukh Omar, Fabio Miranda, Tanvi Bhatt

**Affiliations:** 1 Department of Physical Therapy, University of Illinois Chicago, Chicago, USA; 2 Department of Computer Science, University of Illinois Chicago, Chicago, USA

**Keywords:** Slip, Trip, Deep learning, Gait detection, Bi-GRU model

## Abstract

Accurate detection of gait events in older adults, particularly during perturbed walking, is essential for evaluating balance control and fall risk. Traditional force plate-based methods often face limitations in perturbed walking scenarios due to the difficulty in landing cleanly on the force plates. Subsequently, previous studies have not addressed gait event automatic detection methods for perturbed walking. This study introduces an automated gait event detection method using a bidirectional gated recurrent unit (Bi-GRU) model, leveraging ground reaction force, joint angles, and marker data, for both regular and perturbed walking scenarios from 307 healthy older adults. Our marker-based model achieved over 97% accuracy with a mean error of less than 14 ms in detecting touchdown (TD) and liftoff (LO) events for both walking scenarios. The results highlight the efficacy of kinematic approaches, demonstrating their potential in gait event detection for clinical settings. When integrated with wearable sensors or computer vision techniques, these methods enable real-time, precise monitoring of gait patterns, which is helpful for applying personalized programs for fall prevention. This work takes a significant step forward in automated gait analysis for perturbed walking, offering a reliable method for evaluating gait patterns, balance control, and fall risk in clinical settings.

## Introduction

Gait event detection is a fundamental component of gait analysis, crucial for the quantitative assessment of spatiotemporal characteristics during both regular and perturbed walking [1, 2]. These characteristics include gait duration, reaction time, step length, and gait stability, which are essential for evaluating gait patterns, assessing balance control reactions, diagnosing gait disorders, and monitoring the efficacy of interventions [3–5]. However, detecting gait events, particularly in perturbed walking scenarios, is one of the most time-consuming processes in gait analysis, underscoring the need for automated solutions to enhance efficiency.

The current gold standard for automated gait event detection typically involves the use of force plates [6]. This method identifies gait events by detecting when the ground reaction force (GRF) values rise above or fall below a predetermined threshold, identifying them as foot touchdown (TD) and liftoff (LO). Accurate detection of these events relies on clean force plate data from both lower extremities. However, clean force plate data is often missing in older adults during perturbed walking due to half landing or cross landing on the force plates, which could greatly affect the detection accuracy, necessitating time consuming manual cross-validation. The limited availability of force plates in many clinical settings restricts the applicability of this method. Therefore, various kinematic-based algorithms have been developed, utilizing data from motion capture systems or wearable sensors such as Inertial Measurement Units (IMUs), to detect gait events. However, kinematic-based methods also have significant challenges for application in resource-constrained clinical settings. These methods often require trained personnel with biomechanical expertise to perform gait event detection, while the clinicians may lack training in gait biomechanics or gait analysis tools. Furthermore, these methods generate large datasets that require complex processing, interpretation, and cross-validation, which are difficult to meet in clinics with limited computational resources and time constraints. Therefore, a cost-effective, easy-to-use, robust, and automation gait-event detection method is urgent for clinical practice.

Studies have demonstrated that automatic gait event detection algorithms based on marker position/velocity achieved high accuracy for regular walking in healthy adults [7–9]. A handful of additional studies have developed algorithms aimed at detecting gait events in pathological gait patterns, which have also demonstrated robust performance, maintaining an average absolute error of less than 40 milliseconds [10, 11]. However, to the best of our knowledge, no automated gait event detection algorithm currently exists for perturbed walking following slip or trip perturbations, which are crucial for assessing balance control and fall risk. Existing methods fail in these perturbed walking scenarios because they rely on assumptions of consistent and predictable gait patterns, which are often violated during perturbations. For instance, slip perturbations can lead to diverse recovery strategies, including recovery steps with toe-contact first, recovery steps with heel-contact first, or even aborted steps without clear toe liftoff [12]. Similarly, trip perturbations can result in highly variable responses such as lowering, elevating, and crossing strategies [13]. Even for the same individual, the recovery strategies could be different from trial to trial. This variability in recovery strategies introduces significant challenges for gait event detection. Previous methods, especially the traditional threshold-based algorithms, cannot handle this unpredictable nature of perturbed gait.

In recent years, deep learning techniques, particularly Recurrent Neural Networks (RNNs), have significantly advanced time series event detection across various fields including acoustics [14–16], seismic analysis [17, 18], manufacturing [19, 20], power systems [21, 22], anomaly detection [23], and medical sciences [24, 25]. Among these developments, the bidirectional gated recurrent unit (Bi-GRU) has emerged as a particularly effective model, outperforming traditional recurrent neural network (RNN) approaches in various studies [14–16, 21, 24, 25]. Building on this foundation, our work aims to develop an automatic gait event detection method specifically for perturbed walking scenarios in older adults, utilizing Bi-GRU models. Here, the *automatic* refers to the ability to detect gait events without requiring manual annotation during data analysis. We conduct a comprehensive analysis using data based on markers, angles, and GRFs to evaluate the feasibility of automatic step-time detection approaches for perturbed walking. This will further enhance operational efficiency by eliminating the need for labor-intensive manual cross-validation for the kinematic approach, which could be easily accomplished with wearable sensors. This paper will detail the methodologies employed, present a comprehensive evaluation of the three models, and discuss potential avenues for further refinement and optimization of our algorithms, thereby contributing to the advancement of automated gait analysis in clinical settings.

## Methods

### Participants

307 healthy older adults (Age: 70 ± 6.3 years; weight: 75 ± 17 kg; height: 1.67 ± 0.11 m; gender: 58% female) from our ongoing NIH project (ClinicalTrials.gov NCT03199729; registration date: 06/19/2017; https://clinicaltrials.gov/ct2/show/NCT03199729) were included in this study. All participants were screened to pass a descriptive questionnaire without self-reported recent neurological, musculoskeletal, or systematic disorders within 6 months. The study was carried out in accordance with the Declaration of Helsinki of 1975, and all participants provided written informed consent which was approved by the Institutional Review Board of the University of Illinois at Chicago (IRB #: 2016 − 0887).

### Experimental setup

All participants experienced at least 10 regular walking, 1 slip, and 1 trip perturbation trials. The first 2 slips (if available), first 2 trips, and 3 random regular walking trials were selected. Although 2 slip trials and 2 trip trials from the same subject were included, the inherent variability caused by the first-trial learning effect minimized the risk of data leakage [26]. Totally, the dataset consists of 927 regular walking, 399 trips, and 529 slip trials. All the trials were collected on a 7-meter walkway. The slip perturbations were triggered by a movable slider with a maximum distance of 75 cm, and trip perturbations were induced by an obstacle device with a height of 8 cm. During regular walking, the movable slider and obstacle device were locked by a pair of electromagnets. During slip trials, the movable slider would be released within 50ms after a participant’s right (slipping) foot was detected in contact with the right platform, detected by the force plates (AMTI, Newton, MA) installed beneath the right platform. During trip trials, the obstacle device was triggered when the vertical ground reaction force (GRF) under the unperturbed (right) limb exceeded 80% of the participant’s body weight after the touchdown of the right foot. Once the plate was unlocked, it could reach its upright position in less than 150ms. This would guarantee that all trips occurred in the late-swing phase. During regular walking, both the movable platform and the trip plate were locked. In all the trials, participants were instructed to walk at their preferred speed and in their preferred manner, and they were told that a slip or trip perturbation may or may not happen during any of the trials.

A full-body safety harness connected by shock-absorbing ropes to a loadcell was used to protect participants from falling and to detect harness-supported body weight (Transcell Technology Inc., Buffalo Grove, IL). The harness enabled participants to walk freely while providing protection against body impact on the floor. Kinematics from a full-body marker set (30 retro-reflective markers) were recorded by an eight-camera motion capture system (Qualysis, Gothenburg, Sweden) at 120 Hz and synchronized with the force plate (AMTI, Newton, MA) data at 600 Hz. Individual markers were initially identified and gap-filled using Qualisys software. Marker data were then analyzed using a custom MATLAB code (MathWorks, Natick, MA, USA) to generate foot, thigh and shank segment angles in sagittal plane. The foot angle was calculated as the angle between the horizontal line and the line connecting toe and heel markers, the thigh angle was calculated as the angle between the horizontal line and the line connecting hip marker and knee marker in sagittal plane, and the shank angle was calculated as the angle between the horizontal line and the line connecting knee marker and ankle marker.

### Data pre-processing

For the *GRF dataset*, GRF in anteroposterior (AP), vertical (VT), and mediolateral (ML) directions within 3 s were used as input observations. The duration contains 2 TD and 2 LO events for the left side (recovery side for slip, perturbed side for trip) on the force plates. However, due to the size limitation of the force plates, the GRF for other steps was not collected, and, therefore, not included in this study. For the *marker dataset*, trajectories of 8 markers (heel, toe, knee, and hip for both limbs) in AP and VT directions within the same duration were used as input observations. These markers were selected according to previous research [27–29]. For the *angle dataset*, trajectories of 6 segment angles (foot, shank, and thigh for both limbs) in the sagittal plane were calculated using the marker coordinates and used as input observations. The GRF was low-pass filtered at 50 Hz using a bidirectional fourth-order Butterworth filter, then downsampled to 120 Hz to match the dimension of kinematic data [30]. The marker and angle data were firstly gap-filled based on the nearest non-missing value (fillmissing function in MATLAB), and then low-pass filtered at 12 Hz using a bidirectional fourth-order Butterworth filter [30].

**Fig. 1:**
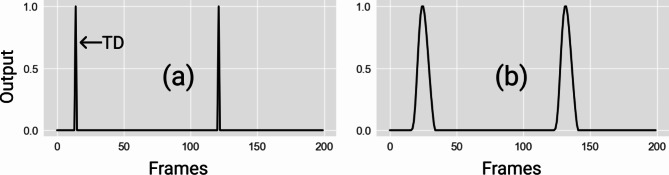
A sample of (**a**) raw 1-D output data and (**b**) output data processed with the HRF

For the output data, TD and LO events were manually detected and cross-validated, for each frame we encode a TD as 1, LO as -1 and a non-event as 0. As a result, each output consisting of 360 frames (120 frames per second) is encoded as a vector of {0, 1, -1}. Figure 1a illustrates instances of TD events, represented by values of 1 amidst zeros. To better map the multivariate time series input data to the 1-dimensional time series output data, we applied a hemodynamic response function (HRF) to the 1D output data (Fig. 1b). By employing the HRF function, we can account for temporal dynamics in the gait data, effectively smoothing the transitions (i.e., 0 to 1) and creating a more robust representation of the event-related signals. This approach offers several benefits. First, it helps capture not only the precise moment of each gait event but also the temporal effects around these events, such as the gradual changes before and after TD or LO. Second, the HRF helps mitigate data imbalance issues during model training. In gait event detection, non-events are more frequent than other events (Fig. 1a), leading to a serious imbalance in the output labels. the HRF helps to normalize the influence of more frequently occurring events compared to rarer ones. Lastly, this approach improves the robustness of the output data, as the HRF-enhanced signals align better with the inherent biomechanical processes underlying gait. It allows the model to consider both immediate and residual effects for gait event detection. The data processing and implementation of the HRF were conducted using customized MATLAB scripts.

### Model architecture

**Fig. 2:**
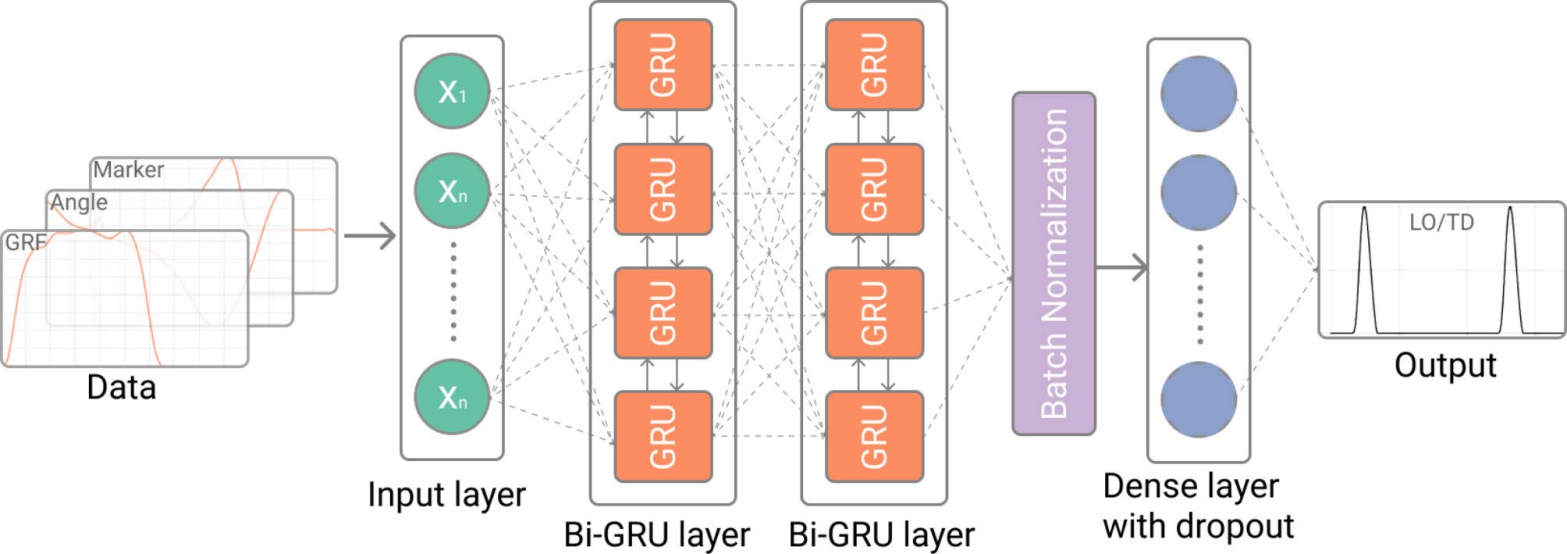
Proposed multi-layer neural network architecture featuring bidirectional GRU Layers

To map the multivariate time series input data to a one-dimensional time series output, we developed and trained a deep learning model featuring Bi-GRU layers, which are well-suited for sequential data modelling. Our network architecture includes two Bi-GRU layers, supplemented by a batch normalization layer to stabilize learning [31], a dropout layer to prevent overfitting [32], and a dense layer for output integration (Fig. 2). Integrating two Bi-GRU layers enhanced our network’s capability to discern complex relationships and deeper features in the data, but adding more than two layers did not improve performance due to shortage of data.

The network utilizes the Adam optimizer [33] and a custom weighted loss function. This loss function, designed to square the differences between predicted and observed outputs, was adapted to emphasize errors proportionally to their importance; observations closer to 1 received higher weights, reflecting their greater significance in the training process. Training was conducted using the standard mini-batch technique with batch sizes of 64. The training was halted if no improvement in the loss function was observed over three consecutive epochs, with a total of 100 epochs planned for the entire training process. The Bi-GRU architecture could capture both forward and backward temporal dependencies in the input data, making it well-suited for time-series modelling of gait events. Compared to LSTMs, Bi-GRUs offer similar performance with reduced computational complexity [34]. While Transformer-based models have shown promise in many domains, they generally require larger datasets for effective training [35], which may not align with our dataset size. Hence, the Bi-GRU is selected in this study.

In the development of recurrent neural networks (RNN), Cho et al. proposed the gated recurrent units (GRU) [36]. The GRU structure is as follows. In the initial state, the output vector starts at zero: $\:{h}_{t}=\:0,t\:=\:0$. Then,

1 $$\:{z}_{t}=sigmoid\:({W}_{z}{x}_{t}+\:{U}_{z}{h}_{t-1}+\:{b}_{z})$$

2 $$\:{r}_{t}=sigmoid\:({W}_{r}{x}_{t}+\:{U}_{r}{x}_{t}+\:{b}_{r})$$

3 $$\:{\overline{h}}_{t}=tanh\:({W}_{h}{x}_{t}+\:{U}_{h}({r}_{t}\odot\:{h}_{t-1})\:+{b}_{h}$$

4 $$\:{h}_{t}=(1-{z}_{t})\odot\:{h}_{t-1}+{z}_{t}\odot\:{\overline{h}}_{t}$$

Where, $\:\odot\:$ defines dot product, $\:{x}_{t}$ is the input vector at time step *t*, $\:{h}_{t}$ is the output vector, $\:{\overline{h}}_{t}$ is the candidate activation vector, $\:{z}_{t}\:$is the update gate vector, $\:{r}_{t}$ is the reset gate vector. $\:W$, $\:U$ are the parameters matrices, and $\:b$ is the parameters vector. The Update Gate ($\:{z}_{t}$) utilizes a sigmoid function to determine how much past information should be retained, thereby influencing the amount of information carried forward. The Reset Gate ($\:{r}_{t}$) decides the extent to which past information is forgotten, enabling the model to eliminate irrelevant data. The Candidate Activation ($\:{\overline{h}}_{t}$) suggests new memory content by combining new input with past information that has been adjusted by the reset gate. Finally, the Final Memory Update ($\:{h}_{t}$) merges the old state and the new candidate activation, as moderated by the update gate, to update the current state effectively.

Contrary to GRU, Bi-GRU is constructed by two unidirectional GRUs facing opposing directions [37]. The calculation of Bi-GRU can be formulated as follows.

5 $$\:{\overrightarrow{h}}_{t}={GRU}_{fwd}\:({x}_{t},\:{\overrightarrow{h}}_{t-1})$$

6 $$\:{\overleftarrow{h}}_{t}={GRU}_{bwd}\:({x}_{t},\:{\overleftarrow{h}}_{t-1})$$

7 $$\:{h}_{t}={\overrightarrow{h}}_{t}\oplus\:{\overleftarrow{h}}_{t}$$

Here, $\:{\overrightarrow{h}}_{t}$ and $\:{\overleftarrow{h}}_{t}$ are the state information of the forward and backward GRU, respectively. $\:{GRU}_{fwd}$ is the forward GRU, and $\:{GRU}_{bwd}$ is the backward GRU. Both GRUs follow the formulation of Eq. (1) – Eq. (4). $\:\oplus\:$ denotes concatenating the $\:{\overrightarrow{h}}_{t}$ and $\:{\overleftarrow{h}}_{t}$.

Following the two Bi-GRU layers, the subsequent steps of our architecture are outlined as follows.

8 $$\:{x}_{norm}=BatchNorm\left(x\right)$$

9 $$\:{x}_{drop}=Dropout\left({x}_{norm}\right)$$

10 $$\:{y}_{t}=Dense({x}_{drop},\:\:V,\:b)$$

Here, $\:V$ and 𝑏 are the weights and biases of the dense layer, respectively. The custom weighted loss function is designed to square the differences between predicted and observed outputs is as follows.

11 $$\:L = \sum {(\lambda {\:_t}.{{\left( {{y_t} - {{\bar y}_t}} \right)}^2})}$$

Here, ​$\:{\lambda\:}_{t}$ is the weight assigned to the observation at time step 𝑡.

For the evaluation and training of our models, we divided each dataset (marker, angle, and ground reaction force) into three distinct parts. Initially, we partitioned the entire dataset into an 80% training set and a 20% test set (train_test_split from sklearn). Subsequently, we further split the training set, allocating 20% of it for validation purposes (set validation_split = 0.2 in model.fit).

To identify gait events from the output of our trained models, we utilized a peak detection algorithm (find_peaks from scipy) configured to detect peaks with required minimal time interval between neighbouring peaks > 250ms and height of peaks > 0.3, default values were used for other parameters (i.e., width = None, prominence = None, rel_height = 0.5). Here, the 250 ms was selected as it reflects the minimum time interval required for sequential gait events to occur under both regular and perturbed walking conditions. According to prior research, the step initiation time or step latency (from perturbation onset to recovery foot liftoff) in perturbed gait is around 300 ms [38], and the whole gait duration would be longer as the swing phase (foot liftoff to its touchdown) is around 100 ms in perturbed walking [39]. Therefore, 250 ms is a reasonable threshold to differentiate distinct gait events. The peak height threshold of 0.3 was chosen to ensure that only peaks corresponding to gait events were detected and filter out noise or low-amplitude fluctuations that do not represent actual gait events. The specific value was empirically determined based on the characteristics of the dataset, ensuring robustness across various trial types, including perturbed walking. Then, we calculated and compared the discrepancies between the detected outputs and the observed outputs across the different models. We trained the model separately for TD and LO detection and detected gait events only on the left side as it was more affected by external perturbation. The model training was conducted in Python using Keras version 2.12, with TensorFlow serving as the backend framework.

### Evaluation of performances

**Fig. 3:**
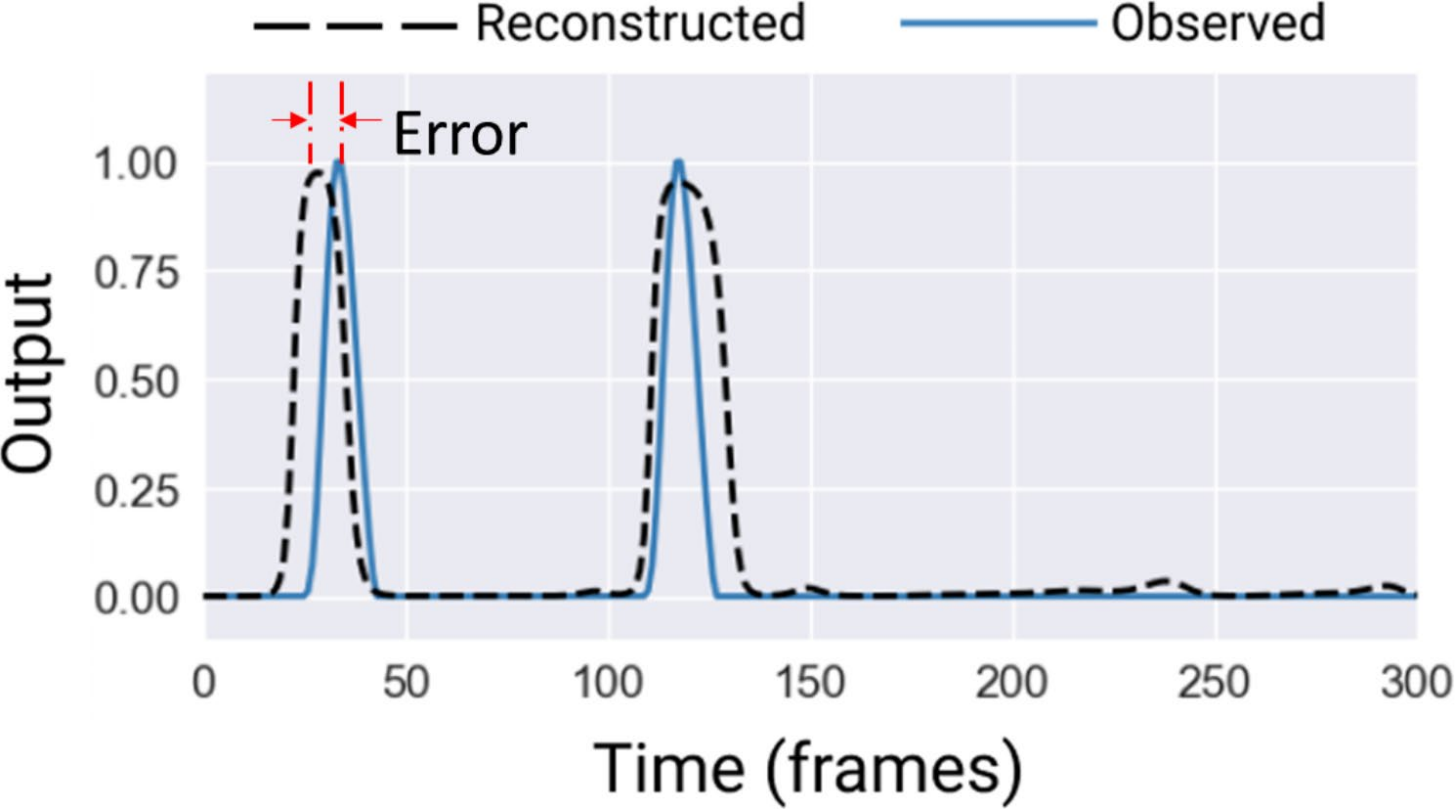
A sample of observed 1D time series output (solid) from the marker-based model for TD detection and reconstructed output (dashed). Here, each peak represents a TD event

The test set was used to assess the performance of various models by measuring the absolute error between the observed output (timing of gait events) and the predicted output, as depicted in Fig. 3. For gait analysis and efficient real-time control of simulation devices such as functional electrical stimulation (FES) systems [40], it is crucial that the target detection time maintains an error margin below 50 ms. Accordingly, a tolerance of 50 ms, equivalent to 6 frames at the given sampling rate, was established as the criterion for calculating the accuracy of detection rate [41]. Additionally, detection accuracy was also calculated using a tolerance of 30 ms [10, 42–44], a threshold previously adopted in similar studies [45, 46].

### Statistical analysis

A two-way ANOVA was conducted to analyze the effect of different models (GRF, angle, and marker) and trial types (regular walking, slip, and trip) on the error of detected gait events. Significant main effects and interactions were followed up with independent t-tests to compare errors across models using different datasets and across different trial types. A significant level of 0.05 was used for all the analyses. All statistical analyses were performed in Python.

## Results

Our results demonstrate that all the models could accurately detect TD and LO events with a mean error of < 21ms (Table 1). Moreover, over 92% of TD events and over 93% of LO events were accurately detected (error < 50ms). Two-way ANOVA comparison across the three different models revealed trial effect (F = 11.5, *p* < 0.001) but not model effect on the error of TD detection. Post-hoc test indicated that slip trials and trip trials showed larger error in detected TD than natural walking trials (*p* < 0.001 for both), and no difference was found between slip trials and trip trials. Although no model effect was found on the error of TD detection, marker-based model showed the smallest error (13.6ms) and the highest detection accuracy (97% <50ms and 90.5% <30ms). According to the performance within different trials (Table 2), TD detection for natural walking showed the smallest error (≤ 13.52ms) and highest accuracy (over 96.9% <50ms and over 90%<30ms). Additionally, the marker-based model showed comparable accuracy (95.1-98.5%) across different types of trials based on the tolerance of 50ms.

**Table 1:** Overall performance of different models for TD and LO detection.

Model	Error (mean ± SD)	% of error < 50ms	% of error < 30ms
GRF-TD	15.94 ± 51.13	92.91%^#^	86.92%
Angle-TD	17.88 ± 52.03	95.02%	86.56%^#^
Marker-TD	13.58 ± 20.31	97.04%	90.46%
GRF-LO	20.54 ± 70.18^#^	93.14%	88.28%
Angle-LO	12.24 ± 32.46	97.55%	92.23%
Marker-LO	9.96 ± 12.89*	98.91%*	94.26%^*^

^*^ best model for each metric, ^#^ worst model for each metric

**Table 2:** Performance of models for TD detection in natural walking, trip and slip trials.

Model	Type	Mean error (ms)	% of error < 50ms	% of error < 30ms
GRF-TD	Nat	11.61	96.89%	93.11%
	Trip	22.72	88.95%^#^	81.4%
	Slip	19.94	87.24%	77.55%^#^
Angle-TD	Nat	13.52	97.97%	90.1%
	Trip	28.61^#^	94%	83.33%
	Slip	18.42	90%	82%
Marker-TD	Nat	10.81^*^	98.46%^*^	94.62%^*^
	Trip	16.56	96%	86%
	Slip	16.67	95.1%	85.78%

^*^ best model for each metric, ^#^ worst model for each metric

For LO detection, two-way ANOVA results showed significant model effect (F = 11.36, *p* < 0.001) and trial effect (F = 4.2, *p* = 0.015) on the error with a significant interaction effect (F = 4.26, *p* = 0.002). Post-hoc test indicated that both marker-based model and angle-based model have a smaller error than the GRF-based model (*p* ≤ 0.003 for both), with no significant difference between the marker-based and angle-based models. However, based on the detection rate, marker-based model showed the best performance with the highest detection accuracy (98.9% <50ms and 94.3% <30ms). Performance within different trials showed that the LO detection for natural walking exhibited the smallest error and highest accuracy for GRF-based model (Table 3). Conversely, the marker-based and angle-based model demonstrated comparable accuracy (95.1-98.5%) across different types of trials.

**Table 3:** Performance of models for LO detection in natural walking, trip and slip trials.

Model	Type	Mean error (ms)	% of error < 50ms	% of error < 30ms
GRF-LO	Nat	14.5	97.06%	94.61%
	Trip	19.86	89.89%	82.98%
	Slip	33.13^#^	88.35%^#^	80.58%^#^
Angle-LO	Nat	10.38	97.64%	93.72%
	Trip	17.46	96.43%	88.69%
	Slip	11.32	98.37%	92.39%
Marker-LO	Nat	10.52	98.65%	92.97%
	Trip	9.09^*^	99.40%^*^	95.78%^*^
	Slip	9.65	98.98%	95.41%

^*^ best model for each metric, ^#^ worst model for each metric

## Discussion

This study developed three automatic gait event detection models utilizing on GRF, angle, and marker data. All these models could accurately detect TD and LO events for both perturbed and unperturbed walking. Our comparative analysis further reveals that, contrary to traditional views which favor GRF-based methods as the gold standard, alternative kinematic approaches utilizing marker and angular data yield promising results. Notably, the marker-based model achieved accuracy > 97% and a mean error < 14 ms in detecting both TD and LO events within datasets containing regular and perturbed walking trials, underscoring the effectiveness of motion data in enhancing automatic gait event detection.

The models developed in our study can significantly reduce engineering costs and increase the accuracy of detected gait events. Traditional methods relying on GRF thresholds are often ineffective for perturbed walking due to varied recovery strategies after external perturbations [12, 13]. The perturbation could additionally introduce noise in the GRF signals. For example, events like the contact between the foot and obstacle, the trigger of perturbation, and the hitting between the slider and blocker can result in a GRF above the threshold, leading to false positives. Therefore, manual gait event detection becomes necessary for the perturbed trials. This process takes over 5 min per gait cycle for pertutrbed walking trials, and cross-validation is necessary to mitigate the effects of human errors and biases on the gait events, which further increase the time consumption. Our models can substantially reduce the time costs by at least two hours per clinic visit for 24 perturbed trials [47] and minimize errors caused by human factors. Although many kinematic-based automatic detection methods were developed, and most of them showed a good accuracy (Precision or F1 score > 90%) of gait-event detection for regular walking in healthy older adults [48, 49], patients with multiple sclerosis [50], Parkinson’s Disease patients [51], and post-stroke patients [50]. However, previous study evaluated different automated event detection algorithms in pathological gait and found that their accuracy is relatively lower in certain abnormal gait scenarios [10]. For example, in gait with transverse plane (rotational) abnormalities, which often occur in slip trials, previous algorithms only detect less than 80% of TD events within four frames (33ms) [48, 52]. Similarly, in gait with foot dragging, which could occur in both slip and trip trials, previous algorithms could only identify 70–89% of LO events within four frames [48, 53, 54]. Moreover, the unpredictable and inconsistent nature of gait patterns (no matter normal or abnormal) during perturbed walking further challenges the reliability of these algorithms. Individual’s gait pattern could change from step-to-step or trial-to-trial. As a result, their accuracy of existing methods in detecting gait events under such conditions is significantly affected.

**Fig. 4:**
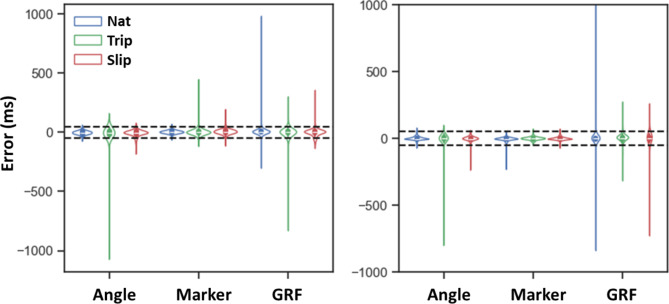
The violin plot of error distributions across all models and trial types for TD detection (left) and LO detection (right). The dashed black lines indicate ± 50 ms threshold of error

Although the GRF-based method was traditionally considered a gold standard for automatic gait event detection [6], in this study, the GRF-based model exhibited the worst performance, particularly for perturbed walking trials, compared to other models. For regular walking, all three models showed comparable detection accuracy (96.9-98.7% for all models in Tables 2 and 3), suggesting that the discrepancy in performance are primarily influenced by walking scenarios. Perturbed walking could introduce significant noise into GRF signals, which likely affect the models’ accuracy. Even if the perturbation occurs during forward walking in our study, individuals may still experience balance loss in the mediolateral direction and pelvis rotation, causing the recovery step to land across the walkway’s midline. This results in increased GRF or abrupt spikes in GRF signals for both limbs at recovery TD, leading to failures in gait event detection for the GRF-based model. In contrast, the marker-based or angle-based models rely on kinematic data, which demonstrate greater resilience to noise in perturbated trials due to their ability to capture overall body movements rather than localized interactions (i.e., points of foot contact). Hence, these data are relatively less affected by the variability of recovery strategies during perturbations. Our results further showed that the performance for angle-based and marker-based models are comparable across different trial types (detection accuracy in Table 3). Given that cross-landing or cross step is common in perturbed walking trials [55], we chose not to exclude such trials to maintain a large sample size for model training. Additionally, our goal is to develop a model with robust generalization capabilities, ensuring reliable gait event detection regardless of cross-landing occurrences.

Besides signal noise, the observed discrepancy in performance among the three models may be influenced by the specific architecture selected in this study. The Bi-GRU architecture was chosen for its ability to capture temporal dependencies in sequential data effectively, but it might not fully leverage the spatial characteristics of GRF data compared to kinematic data. While architectures that combine RNNs with convolutional layers or those specifically designed to process multi-dimensional time-series data (e.g., CNN-RNN hybrids) might better capture the rich spatiotemporal features inherent in GRF data [56]. Future work could explore alternative architectures better suited to GRF data to further improve the model’s performance.

Our methods resulted in a larger standard deviation of errors (> 32 ms) for the angle-based and GRF-based models. This variability can be attributed to the detection of extra peaks in the estimated 1D output data. These extra peaks could lead to substantial errors, exceeding 500 ms (see Fig. 4), which significantly increases both the mean value and standard deviation of the absolute error. In slip trials, these extra peaks were often misidentified as a TD event due to aborted stepping, a scenario in which the foot is not clearly lifted off and foot dragging may occur after the LO phase [12]. Implementing a more refined peak detection algorithm based on peak properties (i.e., peak height, peak width, plateau size, and peak distances) could mitigate this issue by more accurately identifying peaks corresponding to actual gait events, thereby improving the overall performance of the detection methods.

Several strategies can be explored to further improve the overall performance of our models. First, expanding the dataset through the collection of more gait data or by employing data augmentation techniques such as noise injection can increase both the volume and diversity of the training data, facilitating the training of deeper networks. Exploring ensemble methods could also improve performance by leveraging the strengths of multiple models [57]. Lastly, we plan to investigate more complex RNN architectures to further refine and strengthen our model.

There are several limitations in this study. First, the models developed in this study were only verified using gait data from healthy older adults, which limits the generalizability of our findings to clinical populations. To explore the effectiveness of these models for different populations, future research should include individuals with pathological gait patterns, such as those with chronic stroke, Parkinson’s disease, or multiple sclerosis. Collecting data from these populations will allow us to refine the model for diverse gait patterns and validate its robustness in real-world clinical settings. Second, the automatic gait event models were only developed and verified for the left limb, which takes the first compensatory stepping after both slip and trip perturbation. The gait events for this limb are more affected by human errors and biases due to the variety of recovery strategies employed by this limb during the first compensatory stepping. Therefore, it is reasonable to postulate that these models should have a higher detection accuracy for the right limb. However, further validation is required to verify this.

## Conclusion

Our study is the first to develop and evaluate automatic gait event models using deep learning methods for perturbed walking. The kinematic-based models could accurately detect over 95% of gait events with an absolute error of < 50 ms for both regular and perturbed walking. The ability to automatically detect gait events during perturbed conditions is crucial for understanding and analyzing balance control mechanisms and reactive strategies for fall prevention [58]. Wearable sensors (i.e., IMU sensor) or computer vision techniques can be integrated into real-time gait analysis using the proposed models. For example, angle-based models can leverage joint angle data captured by IMUs to provide real-time detection of gait events like TD and LO. By implementing these models in embedded systems, wearable devices can monitor gait patterns continuously, providing alerts for potential fall risks or abnormal gait patterns. This integration could also help fall prevention programs by providing real-time feedback, it can identify deviations from desired movement patterns or ineffective reactive strategies, thereby correcting gait pattern or reactive actions to improve stability and reduce fall risks. Additionally, long-term monitoring enables the development of tailored adjustment programs that adapt to an individual’s progression over time, ensuring personalized and effective intervention.

## Data Availability

No datasets were generated or analysed during the current study.
